# Primary Hydatid Cyst of the Trapezius Muscle: An Unusual Location and Review of the Literature

**DOI:** 10.7759/cureus.56141

**Published:** 2024-03-14

**Authors:** Ousama Jelti, Oussama El Alaoui, Adnane Lachkar, Najib Abdeljaouad, Hicham Yacoubi

**Affiliations:** 1 Department of Orthopedics and Traumatology, Mohammed VI University Hospital, Oujda, MAR; 2 Faculty of Medicine and Pharmacy, Mohammed First University, Oujda, MAR; 3 Department of Orthopedics and Traumatology, Centre Hospitalier Universitaire (CHU) Mohammed VI, Oujda, MAR

**Keywords:** unusual localization, surgery, imaging, trapezius, hydatid cyst

## Abstract

Hydatidosis is a cosmopolitan anthropozoonosis common to humans and many mammals, caused by the development in the body of a dog tapeworm called *Echinococcus granulosus*. As accidental intermediate hosts, humans contract the infection either directly through contact with dogs or indirectly by ingesting contaminated food. They represent an epidemiological dead-end. Hydatid cysts are typically associated with the liver and lungs and, more rarely, with bones, the brain, eyes, heart, kidneys, and spleen. We present an unusual case of a hydatid cyst located in the trapezius muscle of a 76-year-old woman. Clinical, biological, and radiological data allow us to evoke the diagnosis and avoid an inopportune puncture. Treatment was exclusively surgical, with the removal of the cyst without cyst breach.

## Introduction

Echinococcosis, also known as hydatid disease, is a form of zoonotic infection caused by the larval development or formation of cysts from the *Echinococcus* genus. The primary hosts of this infection are dogs and foxes, while sheep act as secondary hosts for the infective larval stage [1]. As accidental intermediate hosts, humans contract the infection either directly through contact with dogs or indirectly by ingesting contaminated food. They represent an epidemiological dead-end [1]. The primary localization of echinococcosis in muscles is exceptionally rare, even in highly endemic countries, accounting for in-between 1% and 5.4% of hydatidosis cases [2,3]. We report an observation of a particular localization of a muscular hydatid cyst (HC).

## Case presentation

**Figure 1:**
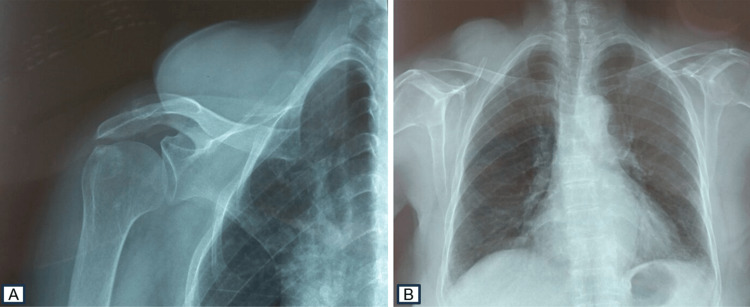
Increased soft tissue contours on the shoulder X-ray with a normal bone skeleton (A, B).

A 76-year-old woman presented a history of a non-painful mass developing on the right shoulder, with a progressive increase in size over the past four years. No other symptoms were associated with this progression. Her medical history was notable for contact with dogs. Upon admission, the physical examination revealed a patient in good overall condition without fever, with a mass on the right shoulder in line with the trapezius muscle, measuring 9x6 cm in diameter, resilient, painless, and mobile in relation to the superficial plane. The mass did not exhibit signs of fluctuation, erythema, bruising, or lymphadenopathy. The standard X-rays revealed a normal bone structure with thickening of soft tissues without calcification (Figure 1).

**Figure 2:**
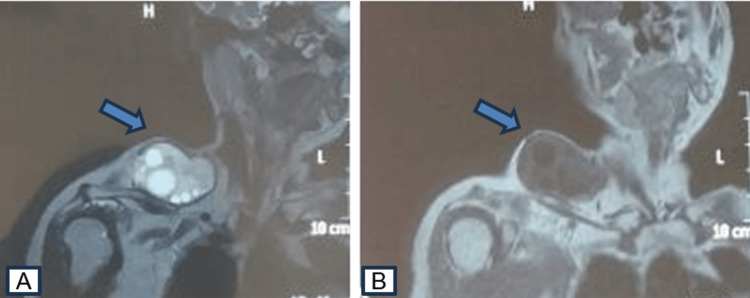
MRI confirming the multilocular appearance of the mass (A, B). MRI: magnetic resonance imaging

Magnetic resonance imaging (MRI) found a well-defined muscular mass located within the trapezius muscle, hypo-intense in T1, and hyper-intense in T2 with a thin and regular wall, not enhanced by contrast media. There were no abnormalities in vascular or bone signal, primarily suggestive of an HC (Figure 2). A thoraco-abdomino-pelvic tomography, performed to detect potential hepatic or pulmonary involvement, showed no secondary localization. Hydatid serology results were negative, and eosinophilic polymorphonuclear cell levels were within normal limits.

**Figure 3:**
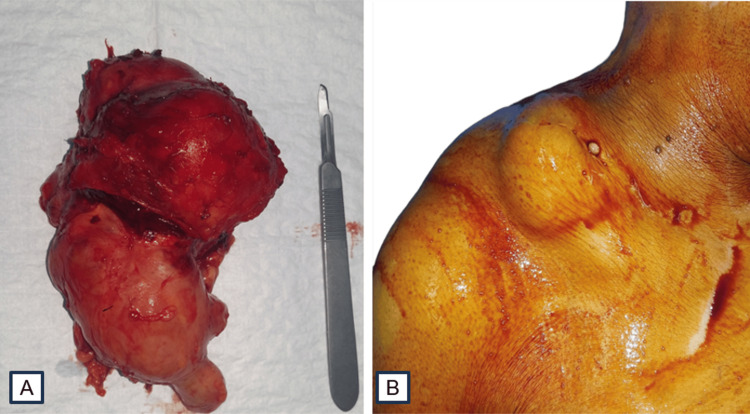
Intraoperative images of the mass (A, B).

The surgical excision was performed under general anesthesia with a direct approach to the trapezius muscle cyst. Complete resection of the cystic mass from the surrounding muscle tissue was carried out without rupture (Figure 3).

The surgical field edges were protected with compresses soaked in hypertonic saline solution. Histological examination of the surgical specimens confirmed the diagnosis of a muscular HC. The immediate postoperative course proceeded without complications, and additional medical treatment with albendazole was initiated on the third day and continued for a total period of six weeks post-surgery to prevent any recurrence. After a three-year follow-up, no local or distant reappearance was observed.

## Discussion

Echinococcosis, a globally prevalent disease, affects both humans and numerous mammals. Dogs, wolves, or certain jackals serve as definitive hosts [1]. Dogs become infested by ingesting contaminated viscera (liver, lung) from sheep, which act as intermediate hosts. The sheep, the main reservoir of the *Echinococcus granulosus* tapeworm, becomes infected by grazing on grass soiled with the dog's feces, containing the eggs of the parasite [4]. Humans, as accidental intermediate hosts, contract the infection either directly through contact with dogs or indirectly by ingesting contaminated food. They represent an epidemiological dead-end [1]. HCs are typically associated with the liver and lungs and, more rarely, with bones, the brain, eyes, heart, kidneys, and spleen [5]. The primary localization of echinococcosis in muscles is exceptionally rare, accounting for 1-5.4% of hydatidosis cases [2,3]. Often, a single muscle is affected, although all muscles can potentially be involved, such as the pectoralis major [2], biceps brachii [6], psoas [7], sartorius [8], quadriceps [9], and gluteus [10]. Although the disease is typically asymptomatic, it can manifest variable clinical signs depending on the size and location of the cyst, as well as the pressure exerted by cyst growth [11].

The clinical manifestation of muscular hydatidosis is limited and characterized by a gradual, painless increase in soft tissue without affecting the patient's overall health, especially in those residing in endemic areas with a history of contact. Occasionally, symptoms of local compression and/or inflammatory signs may be observed, simulating a hematoma, abscess, or malignant tumor [9,12]. The diagnosis of echinococcosis primarily relies on the patient's medical history, findings from physical examination, radiological images, aspiration procedures, and serological analyses.

Conventional radiography often yields normal results. Some cases of aged cysts may reveal intracystic calcification, primarily excluding bone involvement. Ultrasound remains the critical examination for diagnosis, with 100% sensitivity in typical cases. However, atypical forms may present, where the lesion can be mixed, solid, or pseudotumoral, with or without internal echogenicity [13,14]. Computed tomography, although it may face diagnostic challenges similar to ultrasound in atypical forms, provides better accuracy in determining local-regional relationships, especially vascular ones [14,15]. MRI stands out as the most valuable tool in exploring hydatid pathology in soft tissues when the sonographic appearance exhibits atypical characteristics. However, its high cost and limited availability are constraints. The diagnosis is facilitated by the visualization of daughter cysts and/or intracystic membranes, as well as by observing a peripheral rim with relative hyposignal on T2-weighted images, which enhances after gadolinium injection. Moreover, MRI allows for a comprehensive analysis of local-regional relationships [14,16].

The diagnostic confirmation is provided by a positive hydatid serology, although numerous false negatives are reported, reaching an estimated 80% according to the series by Lamine et al. [16]. It is important to note that eosinophilia is not specific, as it is observed in various parasitic infections [17].

The management of muscular HCs relies on surgical intervention. A cautious surgical approach is necessary to avoid accidental cyst opening. It is crucial to protect the surgical field with hypertonic saline from the beginning of the procedure. Although complete excision with total pericystectomy is considered the ideal procedure, this option is not always feasible in cases where the cyst is compromised, deep-seated, or in contact with vessels [15]. Medical treatment with imidazoles plays a limited role in managing muscular cysts. It is prescribed as an adjunct to surgery in cases of recurrence, inoperable cysts, or multiple HCs (hydatidosis). This approach is adopted as a precaution to prevent the possibility of overlooking small undetected cysts during surgery or contaminating the surgical field during cyst excision, with the goal of preventing recurrences [15,18]. Innovative approaches have been recently introduced. In their study, Ormeci et al. [9] detail the treatment of five muscular HCs in three patients, employing a puncture-aspiration-injection method with alcohol and sclerosing agent. They achieved promising results and concluded that this technique stands out for its simplicity, reproducibility, and cost-effectiveness, with straightforward postoperative outcomes. It thus emerges as a surgical alternative for specifically selected patients [9,19].

## Conclusions

Our study highlights an exceptionally rare localization of the primary echinococcal cyst, observed in the trapezius muscle. It underscores the crucial importance of suspecting hydatid disease in the context of musculoskeletal cystic lesions, particularly in endemic geographical areas. Preoperative diagnosis can be challenging, given the complexity of clinical presentations. However, including hydatid disease in the initial differential diagnosis can contribute to preventing serious complications, such as anaphylactic reactions, and reducing the risk of potentially deadly recurrences.
